# BioCurve Analyzer: a web-based shiny app for analyzing biological response curves

**DOI:** 10.1186/s13007-025-01372-x

**Published:** 2025-04-27

**Authors:** Zenan Xing, James Eckhardt, Aditya S. Vaidya, Sean R. Cutler

**Affiliations:** 1 Botany and Plant Sciences, University of California, Riverside, CA 92521 USA; 2 Institute for Integrative Genome Biology, University of California, Riverside, CA 92521 USA; 3 Present Address: HydroGreen, Sioux Falls, SD 57107 USA; 4 Present Address: MilliporeSigma, Temecula, CA 92590 USA

**Keywords:** Dose–response, Time-to-event, Biphasic curve, ED_50_, EC_50_, T_50_, Shiny, R

## Abstract

**Background:**

Dose–response and time-to-event data are common in enzymology, pharmacology, and agronomy studies. Diverse biological response curves can be generated from such data. The features of these curves can be elucidated through parameters such as ED_50_ (the effective dose that gives 50% of the maximum response) and T_50_ (the time required to reach 50% of the maximum response). Properly estimating these parameters is crucial for inferring the potency of compounds or the relative timings of biological processes.

**Results:**

We present an open-source Shiny application, BioCurve Analyzer, that simplifies the process of inferring ED_50_ and T_50_ parameters from response curves exhibiting various patterns, including classic monotonic sigmoidal curves and more complicated biphasic curves. BioCurve Analyzer provides access to several packages and commonly used models for characterizing response patterns, assists users in identifying the models that best describe their data, and includes options for inferring ED_50_ values on both sides of biphasic curves. BioCurve Analyzer also facilitates the visualization of response patterns and allows users to customize their final graphical representation to deliver publication-quality graphs of the data.

**Conclusion:**

BioCurve Analyzer integrates multiple R packages in an easy-to-use web-based interface to facilitate dose–response and time-to-event analyses.

**Supplementary Information:**

The online version contains supplementary material available at 10.1186/s13007-025-01372-x.

## Background

Dose–response (DR) data and time-to-event (TE) data are frequently encountered in plant biology studies as they provide valuable insights into how plants respond to treatments and interact with the environment. Dose–response data measures the relationship between the concentrations of chemicals and the resulting physiological or biochemical responses in plants. Similarly, time-to-event data tracks the occurrence of biological events, such as germination, flowering, and survival under treatments. Both data types capture the behavior of the plants under stress across varying doses and timeframes.

Metrics such as effective doses derived from these data are particularly useful, with the ED_50_ representing the dose–response data. The ED_50_ stands for the dose that gives half of the maximum response, such as the enzyme activity; it is referred to as the EC_50_ (effective concentration) or IC_50_ (inhibitory concentration) in different scenarios. This metric is classic and important in assessing new herbicides or other agrochemicals, as it allows researchers to determine the effective concentration needed to achieve the desired effects [1]. Likewise, specific quantiles are essential for time-to-event studies, such as T_50_, which represents the time that gives half of the maximum response, such as the germination rate. These criteria are frequently used in screening processes as they provide a standardized means of comparing the efficacy of different chemicals or various growth conditions. Thus, it is essential to estimate these values accurately so that scientists can make informed decisions in their research, such as which compounds may be worth further investigation.

Various tools are available to estimate the ED_50_ values from dose–response data, such as GraphPad (https://www.graphpad.com/), MS Excel, and Cheburator [2]. However, all of them have limitations. For instance, the dose should be log-transformed, which leads to the exclusion of the response at dose zero. In addition, these programs often require significant manual effort, making them unsuitable for efficiently analyzing large datasets. As the open-source, robust statistical environment R has developed, a package called *drc* was introduced by Ritz et al. in 2015, offering significant advantages for pharmacological research [3]. To make the R packages more straightforward, web-based tools using Shiny provide a more accessible platform for researchers, such as Autoplate [4], IncucyteDRC [5], SiCoDEA [6], REAP [7], DRomics [8], GRcalculator [9], BMDx [10], et al. These provide a more user-friendly interface for data analysis across various fields, but they are designed for one specific experiment, which makes it difficult for bench workers to apply them to their own research. Furthermore, most of the apps are only available for estimating one type of ED_50_ value or have rigid restrictions on the models available for analysis, making it challenging to analyze biphasic curves, such as bell-shaped curves. Therefore, it is beneficial to develop a more versatile application that is robust enough to analyze dose–response data from a broader range of studies.

When estimating T_50_ values from time-to-event data, one conventional way is to analyze the cumulative proportions of events that occurred. In germination studies, researchers typically compute the germination rate at the end of each time interval, treat this as a continuous variable, and estimate T_50_ values in a way similar to ED_50_ estimation in dose–response studies [11–13]. However, this approach overlooks the uncertainty inherent in the time-to-event data. As censored data, only intervals of possible values can be measured. For example, in germination assays, only the number of germinated seeds during a series of time intervals can be recorded, but the precise timing of the germination is unknown. Neglecting this feature may result in inappropriate estimates and make the final conclusions misleading in the studies. To address this, Onofri et al. developed another R package called *drcte*, an extension of the *drc* package, along with a well-established workflow for analyzing time-to-event data[13]. However, there is no web-based application available for this package, which makes it less accessible and practical for bench researchers.

Here, we introduce BioCurve Analyzer, a Shiny application designed to provide a more intuitive and user-friendly experience for analyzing both dose–response and time-to-event data. It facilitates the generation of biological curves for both data types, and enables the estimate of both absolute and relative ED_50_ or T_50_ values from these curves. Furthermore, this application incorporates three published methods for estimating ED_50_ values and nonparametric models for estimating T_50_ values, enhancing its robustness and flexibility to accommodate diverse data patterns. As a result, it is a versatile tool that caters to a broader research community.

### Implementation

The application is written in R, and the implementation requires R version 4.4.1 or higher and several R packages. The *shiny* [14], *shinyjs *[15], *shinyalert *[16], *shinycssloaders *[17], *shinyhelper *[18], and *bslib *[19] enabled and customized the shiny application to make it more interactive and user-friendly. The *tidyverse *[20], *purrr *[21], *broom* [22], *bigsnpr *[23], and *openxlsx *[24] packages are required to load and pre-process the input data. The *drc *[3], *drcte *[13, 25], and *aomisc *[26] packages are used for the data fitting and model selection in the dose–response data analysis. The *scales* [27], *car* [28]*,* and *stats* [29] packages enable the statistical tests for assessing the best-fit models. To generate the figures and the report, *ggplot2* [30], *ggthemes *[31], *ggpubr *[32], *cowplot *[33], *extrafont *[34], *rmarkdown *[35–37], *knitr *[38–40], *DT* [41], *kableExtra *[42], and *rlist *[43] were used. To generate and import the citations, I used *grateful* [44].

The source code for BioCurve Analyzer, along with the data utilized in this paper, is available via the GitHub repository (https://github.com/ZenanXing/BioCurve-Analyzer.git). The application can be launched on any system that has RStudio installed or available online at the Shiny web server (https://zenanx.shinyapps.io/biocurve-analyzer/).

## Results and discussion

### Main features and functionalities

**Fig. 1:**
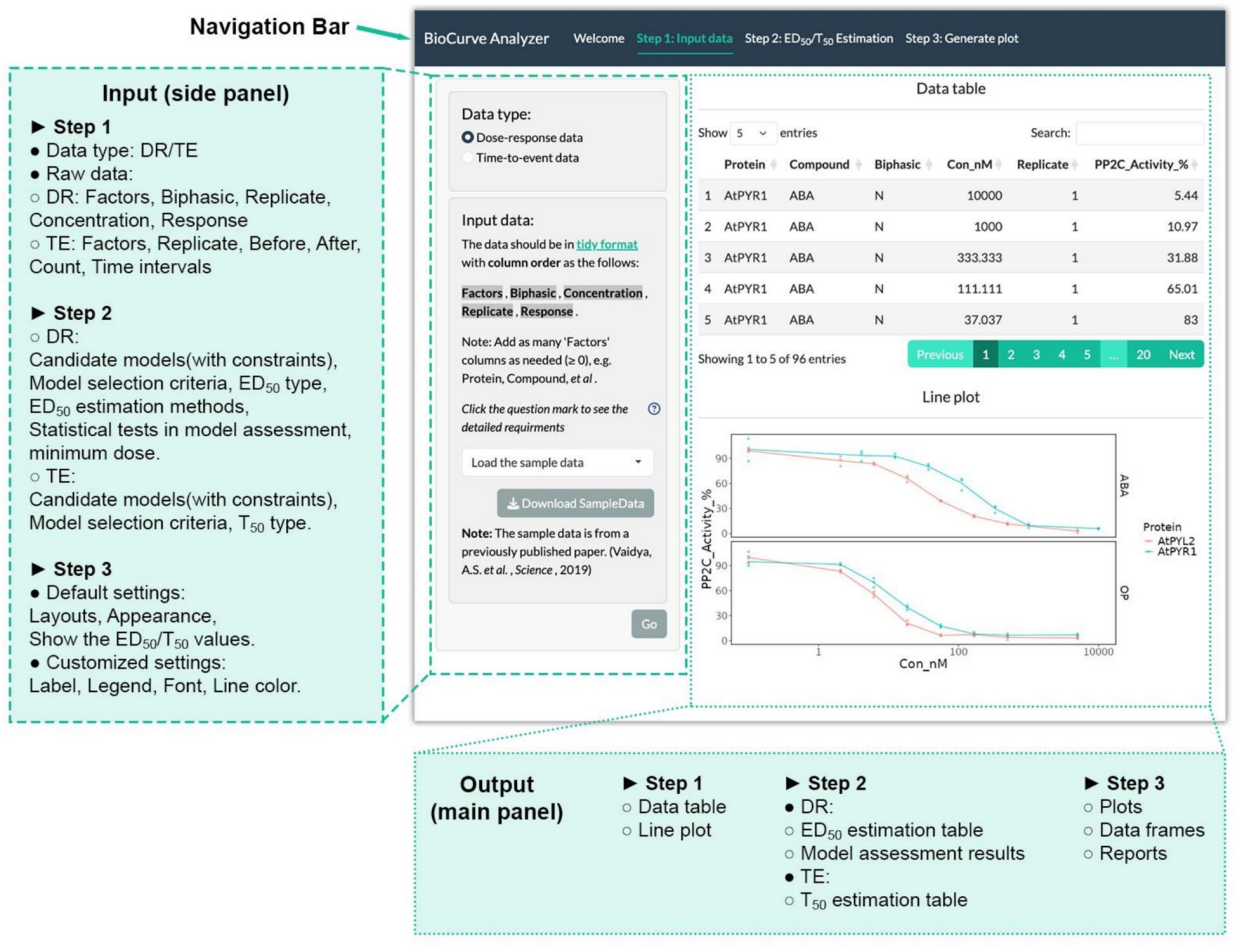
Layout and main features of BioCurve Analyzer. The options in each step to analyze those two data types are slightly different, as illustrated in the figure. DR and TE are short for data types: *DR* stands for Dose–Response, and *TE* stands for Time-to-Event

This application is developed in a tabular fashion. It has three main functionalities available in different tabs: data input, ED_50_/T_50_ estimation, and plot generation, which instructs the user to get the ED_50_/T_50_ and generate the final plot step by step. Upon selecting each tab, the users can specify input values in the side panel and view the corresponding results in the main panel on the right (Fig. 1).

#### Data input tab

To use the application properly, users need to provide the raw data in a tidy format containing essential information such as factors in the experiment, replicates, concentrations/time intervals, and response/counts. The data can be provided as an Excel file or by simply pasting the data, a method that is adopted from a previously published app [45]. After uploading the raw data, the data could be viewed both as a table and a simple line plot in the main panel.

#### ED_50_/T_50_ estimation tab

The ED_50_ and T_50_ can be estimated using the best-fit model in the second tab. The general procedure of estimating both values is the same and involves two main steps. First, it is necessary to identify appropriate models to describe the data. Users are encouraged to empirically select a model based on their experimental objectives to avoid overfitting and ensure consistency in analysis. However, when the underlying mechanisms are complex and unknown, it is advisable to evaluate a set of candidate models and select the one that best fits the data. To assist with this process, the app provides some guidance for selecting candidate models, as different models are suitable for curves with varying shapes. If multiple models are considered, the users are required to select the criteria for determining the best-fit model. The app incorporates model selection metrics commonly used, such as Akaike Information Criterion (AIC) and Bayesian Information Criterion (BIC), which are implemented in the *drc* package. The selected best-fit model is displayed in the main panel.

**Fig. 2:**
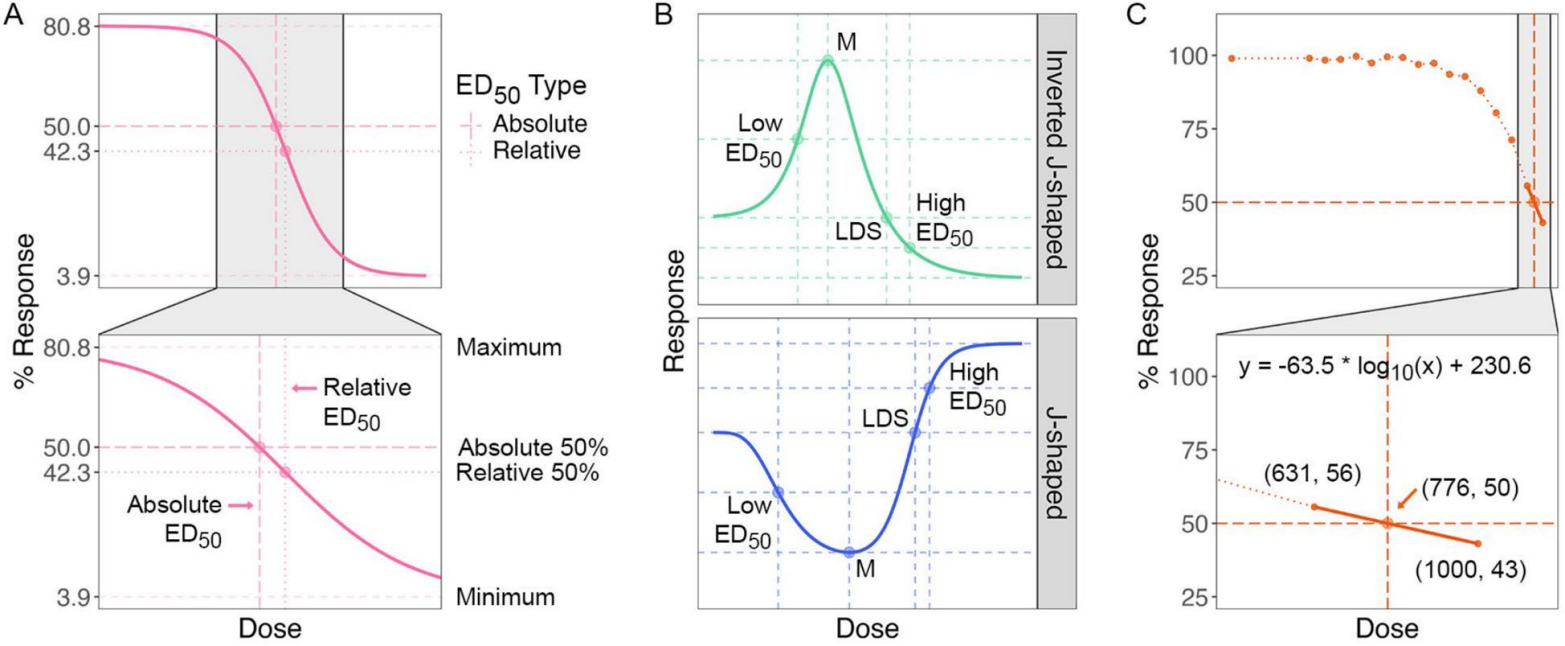
Estimation of ED_50_ from dose–response curves. **A**. The comparison of absolute and relative ED_50_. The relative 50% response and the corresponding relative ED_50_ values are indicated in dotted lines, while the absolute 50% response and the corresponding absolute ED_50_ values are shown in dashed lines. **B.** Parameters estimated from the biphasic curves. The Low/High ED_50_, LDS (limiting dose for stimulation/inhibition), and M (maximum simulation/inhibition) and the corresponding responses are indicated as dashed lines. **C**. Reed-and-Muench method in estimating absolute ED_50_ values. The estimated ED_50_ and the corresponding response are presented as dashed lines in the figure. The coordinates of the ED_50_ and the two data points that bracket the ED_50_ value are shown in the adjacent parentheses, and the function for the linear regression of the two points is displayed. The curves in this figure are generated using simulated data

The next step is to determine whether relative or absolute ED_50_/T_50_ values are more appropriate for the specific analysis. Both relative and absolute ED_50_ values are essential in dose–response studies and have been well-established by the community [46, 47], and the estimates of both types of ED_50_ values for a dose–response curve are illustrated in Fig. 2A. Relative ED_50_ is the dose that achieves 50% of the maximum response relative to the minimum (vertical dotted lines), while absolute ED_50_ is the dose that produces exactly a 50% response (vertical dashed lines). Absolute ED_50_ values are only meaningful when the data are normalized against a control treatment. As a closely related concept, T_50_ values can be inferred from the time-to-event studies. Absolute T_50_ is calculated based on the entire sample, while relative T_50_ is calculated for the fraction of individuals that have experienced that event of interest [13]. For example, in germination assays, if some seeds fail to germinate by the final time interval, it is suggestive to calculate absolute T_50_, as it considers all the tested seeds. However, the relative T_50_ remains valid if independent data confirm that the experiment has run long enough to accurately capture the maximum germination rate, meaning that the ungerminated seeds will not germinate.

Once these steps are complete, the app generates a table containing the estimation of ED_50_/T_50_ values, along with associated statistics such as standard error and confidence interval, which are displayed in the main panel. It should be noted that predicting the behavior of curves beyond the tested dose range or time frame carries inherent risks. Therefore, the app only generates the ED_50_/T_50_ values within the dose range or the time frame provided by the user. To ensure accurate results, users are encouraged to use a broader dose range or time frame to capture the full curve.

Due to the inherent difference between dose–response and time-to-event data, the candidate models used for estimating these values are different. The candidate models for each data type, as well as the additional features available for dose–response data, are outlined in the following sections.

##### Candidate models for each data type

Numerous models have been developed to fit the dose–response data and time-to-event data with diverse trends. It is critical to benchmark these models, particularly to provide suggestions for selecting the best model used to fit the data in terms of accuracy of estimating the effective dose or quantile. The most frequently used models are available in our application and are listed in Supplementary Table 1. Users may select a set of candidate models and allow the app to automatically determine the best-fit model for their data. By default, the app selects all the applicable models, enabling it to identify the best-fit model across a wide range of scenarios. However, this may result in overfitting, so it is advisable for users to select one specific model empirically based on their experimental aims.

The selection of models for dose–response curves largely depends on the shape of the curve [48]. For monotonic curves, the log-logistic models with four parameters are often used to describe the typical symmetrical dose–response curves [48], while the one with five parameters and both Weibull I and Weibull II models are more suitable in describing asymmetric dose–response curves [48, 49]. For biphasic curves, the Brain-Cousens and Cedergreen-Ritz-Streibig models [50, 51] are provided in the app, as they are specifically developed to handle such complex trends.

When analyzing the time-to-event data, the app offers both parametric and nonparametric models. The parametric models include the four-parameter log-logistic model, the Weibull model, and the log-normal model [48]. For nonparametric modeling, the app includes the NPMLE model (nonparametric maximum likelihood estimator) [52] and the KDE model (kernel density estimator) [53], which are well-suited for capturing complex or atypical patterns in the data [13].

##### Three methods in estimating ED_50_

We incorporate three established methods for estimating the ED_50_ values from dose–response data: the Ritz-Gerhard method, the Serra-Greco method, and the Reed-and-Muench method, each named after their respective developers [3, 10, 54]. The key distinctions among these methods are summarized in Table 1. Users can select the most suitable method according to their experimental design and requirements.

**Table 1:** The differences between the ED_50_ estimation methods

Name	Model	Method	Dose-related Output	Monotonic		Biphasic		
				Relative	Absolute	Left		Right
						Relative	Absolute	Relative
Ritz-Gerhard	Non-linear	Delta	Estimate, SE/SD, CI	√	√			√
Serra-Greco	Non-linear	Interpolation	Estimate, CI	√	√	√	√	√
Reed-and-Muench	Linear	Interpolation	Estimate, SE/SD, CI		√		√	

SE/SD and CI stand for standard error/standard deviation and confidence interval, respectively. The last five columns indicate the availability of the three methods based on the given curve shape and ED_50_ type

The curves generated from the time-to-event data are inherently monotonic, as they represent the probability of an event occurring or not occurring up to a certain time. For instance, in the germination assay, the germination rate either remains constant or increases over time but never decreases. In contrast, dose–response studies may produce biphasic or hormetic curves, which contain a rising phase and a falling phase. These curves can be observed in herbicide studies, where certain herbicides stimulate growth at low concentrations but inhibit it at high concentrations, leading to inverted J-shaped curves (top panel in Fig. 2B) [55].

The default method for ED_50_ estimation in the app is the Ritz-Gerhard method, which uses *drc’s* [3] delta method to infer ED_50_ values and their standard errors. This method is best suited to standard sigmoidal or monotonic dose–response curves but not well-suited to biphasic curves. From biphasic curves, additional informative dose-related metrics can be inferred, M, LDS, ED_50_, and f, where M is the dose that elicits the maximum stimulation/inhibition, LDS is the limiting dose for stimulation/inhibition, corresponds to the dose that is equal to untreated control values in the second phase of the curve, and f is a measure of the hormetic-effect. In a biphasic curve, there are two ED_50_ values, one for the left phase (low ED_50_) and one for the right phase (high ED_50_) (Fig. 2B).

Currently, the Ritz-Gerhard method reports only the high ED_50_ values, which limits its applicability for such curves. To address this limitation, we introduced the Serra-Greco method. Serra et al*.* developed an interpolation-based approach to estimate the ED_50_ values in biphasic transcriptomic dose–response curves. This method has been adapted in the app to infer both low and high ED_50_ values for J- and inverted J-shaped curves; this can be selected in the ED_50_ estimation method menu.

Finally, for incomplete dose–response curves that cannot be fit into available models, we implemented the Reed-and-Muench method. This method estimates absolute ED_50_ values using linear interpolation between the two data points that bracket the ED_50_ [54, 56] (Fig. 2C). This approach is particularly useful when the data are insufficient to construct a complete dose–response curve or when fitting a model is not feasible.

##### Statistical tests for model assessment

Another advantage of our app in analyzing the dose–response data is the implementation of statistical tests for the model assessment. To determine whether the best-fit model is appropriate for the dose–response analysis, four statistical tests were introduced in the analysis: lack-of-fit test, Neill's test, no-effect test, and the “parameters ≠ 0” test. They have been frequently used in the model assessment in the dose–response analysis [3, 57]. In general, both the lack-of-fit test and Neill's test are ANOVA-based tests. The lack-of-fit test is a classic test for model assessment, while Neill's test is an enhanced version that can also be applied when there are no replicates. The no-effect test assesses whether there is any dose–response relationship. The “parameters ≠ 0” test examines each parameter in the model individually. Incorporating all tests can help provide a more comprehensive understanding of their model list.

#### Plot tabs

In the plot tab, the curves will be generated using the models selected in the previous step. The estimated ED_50_/T_50_ and the corresponding confidence intervals are shown as the dashed lines in the plot. The application allows the users to modify the layout and the appearance of the figure in this tab. Further customization in different aspects of the figure, such as legend, fonts, and line colors, is also available in the app. Both the tables and the figures are downloadable. If required, users may download the report to get the code used for plotting and modification.

## Application demonstration

To illustrate the functionality of this shiny app, we analyzed previously published dose–response and time-to-event data sets [58–60].

We used BioCurve Analyzer to plot previously published [58, 59] dose–response data generated using receptor/ligand-mediated PP2C (type II C protein phosphatase) inhibition assays. In these assays, abscisic acid receptor agonists bind to the receptor and inhibit PP2C phosphatase activity in a dose-dependent manner, and antagonists allow recovery of PP2C activity in the presence of saturating levels of an agonist [58, 59]. The data analyzed are for the agonists AMF4 (abscisic acid mimic-fluorine derivative 4) and OP (opabactin) and antagonists PANMe and ANT (antabactin) using a wheat ABA receptor TaPYL7 [61]. PANMe was included because it possesses a biphasic bell-shaped dose–response curve that allows us to illustrate BioCurve Analyzer’s ability to infer ED_50_ values within the first phase of a biphasic curve, a function absent from current dose–response curve analysis packages. We note that users must specify if curves are monotonic (i.e. sigmoidal) or biphasic (i.e. inverted J- or J-shaped) in their input dataset to access this functionality.

**Fig. 3:**
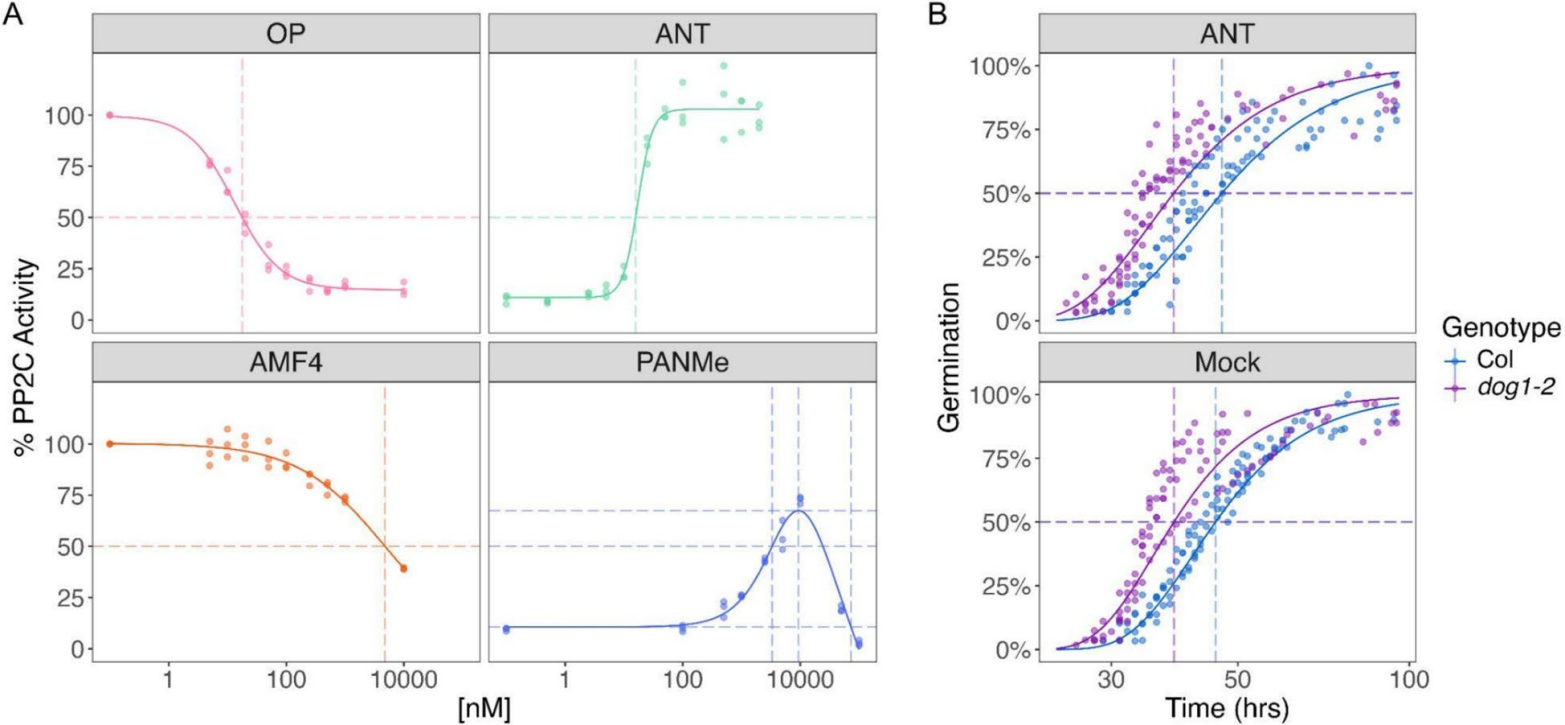
Plots generated by BioCurve Analyzer. **A.** ED_50_ estimation for dose–response data. Dose–response curves and the absolute ED_50_ values of ABA agonists and antagonists. The estimated effective doses using the Serra-Greco method and the corresponding responses are presented as dashed lines. **B**. T_50_ estimation for time-to-event data. The 50% response and the corresponding T_50_ values are indicated as dashed lines. All the curves are generated using the data published in the previous papers [58–60]

In this study, since the activity of PP2C is normalized to that in the absence of any chemicals, it is more appropriate to estimate the absolute ED_50_ values. The generated dose–response curves output by BioCurve Analyzer for the agonist dataset are shown in Fig. 3A, and ED_50_ and statistical tests for model fits are provided in Supplementary Table 2. Given the advantage of the Serra-Greco method in analyzing the biphasic curves, the effective doses shown in the figure are estimated using this method. The input data were specified as monotonic for OP, AMF4, and ANT; and biphasic for PANMe. BioCurve Analyzer’s parameters were set to fit the data to four-parameter log-logistic models for the monotonic curves, while the Cedergreen-Ritz-Streibig model for the biphasic curve, which the app accomplishes using *drc*. As previously described, the inferred ED_50_ of OP is lower than that of AMF4 [58], and the ED_50_ of ANT is lower than that of PANMe [59].

Statistical analysis for the model assessment suggests the best-fit models effectively characterize the data, as almost all the tests showed significant results. Notably, the PANMe’s hormetic effect factor, f, is significantly different from zero, as expected for a biphasic curve (Supplementary Table 2–1). The advantage of the Serra-Greco method in inferring values from biphasic curves is evident in analyzing the PANMe data. This method is capable of generating valid ED_50_, LDS, and M values, which the Ritz-Gerhard method is unable to accomplish (Fig. 3A, Supplementary Table 2–4 and 2–5). For the monotonic curves, the estimated ED_50_ values obtained through both the Ritz-Gerhard and Serra-Greco methods are nearly identical, accompanied by comparable confidence intervals (Supplementary Table 2–2 and 2–3). We note that results derived using the Reed-and-Muench method yield comparable ED_50_ values (Supplementary Table 2–6).

A published time-to-event dataset investigating the role of the gene *Dog1* (*Delay of germination 1*) and the chemical ANT on *Arabidopsis* seed dormancy [60] was used to illustrate the time course data analyses with BioCurve Analyzer (Fig. 3B). The estimated T_50_ values, calculated using *drcte*, are provided in Supplementary Table 3; the generated curves are presented in Fig. 3B. The inferred T_50_ values for the genetic and chemical treatments are consistent with previous descriptions [60].

## Conclusion

BioCurve Analyzer is capable of analyzing both dose–response data and time-to-event data. It provides a user-friendly interface to illustrate the ED_50_/T_50_ estimation procedure in a stepwise manner and allows users to get both absolute and relative values. When estimating the ED_50_ values from dose–response data, the app provides three well-established methods, which gives the users more valid results under different scenarios. In addition, the biological examples illustrate that the BioCurve Analyzer can provide accurate ED_50_/T_50_ estimations, which are valuable for various applications, such as comparing the potency of agrochemicals and revealing the role of key players in biological pathways.

## Supplementary Information

Supplementary material 1

## Data Availability

The data utilized and generated in this paper are available via the GitHub repository (https://github.com/ZenanXing/BioCurve-Analyzer.git).

## Abbreviations

DR: Dose–response
TE: Time-to-event
ED_50_/IC_50_: Effective dose/inhibitory concentration which gives a 50% response in dose–response data.
T_50_: Time which gives a 50% response in time-to-event data.
LDS: Limiting dose for stimulation/inhibition
M: Maximum simulation/inhibition
NPMLE: Nonparametric maximum likelihood estimator
KDE: Kernel density estimator
PP2C: Type II C protein phosphatase
ABA: Abscisic acid
ANT: Antabactin
AMF4: Abscisic acid mimic-fluorine derivative 4
PANMe: PYLs pan antagonist

## References

[1] Jones G. Fitting and handling dose response data. J Comput Aided Mol Des. 2015;29:1–11. 24980646 10.1007/s10822-014-9752-0

[2] Nevozhay D. Cheburator software for automatically calculating drug inhibitory concentrations from in vitro screening assays. PLoS ONE. 2014;9: e106186. 25184280 10.1371/journal.pone.0106186 PMC4153570

[3] Ritz C, Baty F, Streibig JC, Gerhard D. Dose-response analysis using R. PLoS ONE. 2015;10: e0146021. 26717316 10.1371/journal.pone.0146021 PMC4696819

[4] Palmer P, Del Rosario JMM, da Costa KAS, Carnell GW, Huang CQ, Heeney JL, et al. AutoPlate: rapid dose-response curve analysis for biological assays. Front Immunol. 2021;12: 681636. 35222351 10.3389/fimmu.2021.681636 PMC8866857

[5] Chapman PJ, James DI, Watson AJ, Hopkins GV, Waddell ID, Ogilvie DJ. *IncucyteDRC*: An R package for the dose response analysis of live cell imaging data. F1000Res. 2016;5:962. 27703665 10.12688/f1000research.8694.1 PMC5031130

[6] Spinozzi G, Tini V, Ferrari A, Gionfriddo I, Ranieri R, Milano F, et al. SiCoDEA: a simple, fast and complete app for analyzing the effect of individual drugs and their combinations. Biomolecules. 2022;12:904. 35883460 10.3390/biom12070904 PMC9313187

[7] Zhou S, Liu X, Fang X, Chinchilli VM, Wang M, Wang H-G, et al. Robust and efficient assessment of potency (REAP) as a quantitative tool for dose-response curve estimation. Elife. 2022. 10.7554/eLife.78634. 35921131 10.7554/eLife.78634 PMC9348845

[8] Larras F, Billoir E, Baillard V, Siberchicot A, Scholz S, Wubet T, et al. DRomics: a turnkey tool to support the use of the dose-response framework for omics data in ecological risk assessment. Environ Sci Technol. 2018;52:14461–8. 30444611 10.1021/acs.est.8b04752

[9] Clark NA, Hafner M, Kouril M, Williams EH, Muhlich JL, Pilarczyk M, et al. GRcalculator: an online tool for calculating and mining dose-response data. BMC Cancer. 2017;17:698. 29065900 10.1186/s12885-017-3689-3 PMC5655815

[10] Serra A, Saarimäki LA, Fratello M, Marwah VS, Greco D. BMDx: a graphical shiny application to perform benchmark dose analysis for transcriptomics data. Bioinformatics. 2020;36:2932–3. 31950985 10.1093/bioinformatics/btaa030

[11] Ritz C, Pipper CB, Streibig JC. Analysis of germination data from agricultural experiments. Eur J Agron. 2013;45:1–6.

[12] Onofri A, Piepho H-P, Kozak M. Analysing censored data in agricultural research: a review with examples and software tips. Ann Appl Biol. 2019;174:3–13.

[13] Onofri A, Mesgaran MB, Ritz C. A unified framework for the analysis of germination, emergence, and other time-to-event data in weed science. Weed Sci. 2022;70:259–71.

[14] Chang W, Cheng J, Allaire JJ, Sievert C, Schloerke B, Xie Y, et al. shiny: Web Application Framework for R. 2024. https://CRAN.R-project.org/package=shiny

[15] Attali D. shinyjs: Easily Improve the User Experience of Your Shiny Apps in Seconds. 2021. https://CRAN.R-project.org/package=shinyjs

[16] Attali D, Edwards T. shinyalert: Easily Create Pretty Popup Messages (Modals) in `Shiny’. 2024. https://CRAN.R-project.org/package=shinyalert

[17] Attali D, Sali A. shinycssloaders: Add Loading Animations to a `shiny’ Output While It's Recalculating. 2024. https://CRAN.R-project.org/package=shinycssloaders

[18] Mason-Thom C. shinyhelper: Easily Add Markdown Help Files to `shiny’ App Elements. 2019. https://CRAN.R-project.org/package=shinyhelper

[19] Sievert C, Cheng J, Aden-Buie G. bslib: Custom `Bootstrap’ `Sass’ Themes for `shiny’ and `rmarkdown. 2024. https://CRAN.R-project.org/package=bslib

[20] Wickham H, Averick M, Bryan J, Chang W, McGowan LD, François R, et al. Welcome to the tidyverse. J Open Source Softw. 2019;4:1686.

[21] Wickham H, Henry L. purrr: Functional Programming Tools. 2023. https://CRAN.R-project.org/package=purrr

[22] Robinson D, Hayes A, Couch S. broom: Convert Statistical Objects into Tidy Tibbles. 2024. https://CRAN.R-project.org/package=broom

[23] Privé F, Aschard H, Ziyatdinov A, Blum MGB. Efficient analysis of large-scale genome-wide data with two R packages: bigstatsr and bigsnpr. Bioinformatics. 2018;34:2781–7. 29617937 10.1093/bioinformatics/bty185 PMC6084588

[24] Schauberger P, Walker A. openxlsx: Read, Write and Edit xlsx Files. 2024. https://CRAN.R-project.org/package=openxlsx

[25] Onofri A. drcte: Statistical Approaches for Time-to-Event Data in Agriculture. 2023. https://CRAN.R-project.org/package=drcte

[26] Onofri A. aomisc: Utilities for stat courses. Github. https://github.com/OnofriAndreaPG/aomisc

[27] Wickham H, Pedersen TL, Seidel D. scales: Scale Functions for Visualization. 2023. https://CRAN.R-project.org/package=scales

[28] Fox J, Weisberg S. An R Companion to Applied Regression. Third. New Delhi, India: Sage; 2019.

[29] R Core Team. R: A Language and Environment for Statistical Computing. Vienna, Austria: R Foundation for Statistical Computing. 2024. https://www.R-project.org/

[30] Wickham H. ggplot2: Elegant graphics for data analysis. New York: Springer-Verlag; 2016.

[31] Arnold JB. ggthemes: Extra Themes, Scales and Geoms for `ggplot2’. 2024. https://CRAN.R-project.org/package=ggthemes

[32] Kassambara A. ggpubr: `ggplot2’ Based Publication Ready Plots. 2023. https://CRAN.R-project.org/package=ggpubr

[33] Wilke CO. cowplot: Streamlined Plot Theme and Plot Annotations for `ggplot2’. 2024. https://CRAN.R-project.org/package=cowplot

[34] Chang W. extrafont: Tools for Using Fonts. 2023. https://CRAN.R-project.org/package=extrafont

[35] Allaire JJ, Xie Y, Dervieux C, McPherson J, Luraschi J, Ushey K, et al. rmarkdown: Dynamic Documents for R. 2024. https://github.com/rstudio/rmarkdown

[36] Xie Y, Allaire JJ, Grolemund G. R Markdown: The Definitive Guide. Boca Raton, Florida: Chapman and Hall/CRC; 2018.

[37] Xie Y, Dervieux C, Riederer E. R Markdown Cookbook. Boca Raton, Florida: Chapman and Hall/CRC; 2020.

[38] Xie Y. knitr A. Comprehensive Tool for Reproducible Research in R. In: Stodden V, Leisch F, Peng RD, (eds). Implementing Reproducible Computational Research. Chapman and Hall/CRC. Boca Raton. 2014.

[39] Xie Y. Dynamic Documents with R and knitr. 2nd ed. Boca Raton, Florida: Chapman and Hall/CRC; 2015.

[40] Xie Y. knitr: A General-Purpose Package for Dynamic Report Generation in R. 2024. https://yihui.org/knitr/

[41] Xie Y, Cheng J, Tan X. DT: A Wrapper of the JavaScript Library `DataTables. 2024. https://CRAN.R-project.org/package=DT

[42] Zhu H. kableExtra: Construct Complex Table with `kable’ and Pipe Syntax. 2024. https://CRAN.R-project.org/package=kableExtra

[43] Ren K. rlist: A Toolbox for Non-Tabular Data Manipulation. 2021. https://CRAN.R-project.org/package=rlist

[44] Rodriguez-Sanchez F, Jackson CP. grateful: Facilitate citation of R packages. 2023. https://pakillo.github.io/grateful/

[45] Spitzer M, Wildenhain J, Rappsilber J, Tyers M. BoxPlotR: a web tool for generation of box plots. Nat Methods. 2014;11:121–2. 24481215 10.1038/nmeth.2811 PMC3930876

[46] Sebaugh JL. Guidelines for accurate EC50/IC50 estimation. Pharm Stat. 2011;10:128–34. 22328315 10.1002/pst.426

[47] Markossian S, Grossman A, Arkin M, Auld D, Austin C, Baell J, et al. Assay Guidance Manual. Bethesda (MD): Eli Lilly & Company and the National Center for Advancing Translational Sciences. 2004. 22553861

[48] Ritz C. Toward a unified approach to dose-response modeling in ecotoxicology. Environ Toxicol Chem. 2010;29:220–9. 20821438 10.1002/etc.7

[49] Gottschalk PG, Dunn JR. The five-parameter logistic: a characterization and comparison with the four-parameter logistic. Anal Biochem. 2005;343:54–65. 15953581 10.1016/j.ab.2005.04.035

[50] Brain P, Cousens R. An equation to describe dose responses where there is stimulation of growth at low doses. Weed Res. 1989;29:93–6.

[51] Cedergreen N, Ritz C, Streibig JC. Improved empirical models describing hormesis. Environ Toxicol Chem. 2005;24:3166–72. 16445100 10.1897/05-014r.1

[52] Fay MP, Shaw PA. Exact and asymptotic weighted logrank tests for interval censored data: the interval R package. J Stat Softw. 2010;36: i02. 25285054 10.18637/jss.v036.i02 PMC4184046

[53] Barreiro-Ures D, Francisco-Fernández M, Cao R, Fraguela BB, Doallo R, González-Andújar JL, et al. Analysis of interval-grouped data in weed science: the binnednp Rcpp package. Ecol Evol. 2019;9:10903–15. 31641444 10.1002/ece3.5448 PMC6802074

[54] Reed LJ, Muench H. A simple method of estimating fifty percent endpoints. Am J Epidemiol. 1938;27:493–7.

[55] Belz RG, Duke SO. Herbicides and plant hormesis. Pest Manag Sci. 2014;70:698–707. 24446388 10.1002/ps.3726

[56] Ramakrishnan MA. Determination of 50% endpoint titer using a simple formula. World J Virol. 2016;5:85–6. 27175354 10.5501/wjv.v5.i2.85 PMC4861875

[57] Ritz C, Martinussen T. Lack-of-fit tests for assessing mean structures for continuous dose-response data. Environ Ecol Stat. 2011;18:349–66.

[58] Vaidya AS, Helander JDM, Peterson FC, Elzinga D, Dejonghe W, Kaundal A, et al. Dynamic control of plant water use using designed ABA receptor agonists. Science. 2019;366:eaaw8848. 31649167 10.1126/science.aaw8848

[59] Vaidya AS, Peterson FC, Eckhardt J, Xing Z, Park S-Y, Dejonghe W, et al. Click-to-lead design of a picomolar ABA receptor antagonist with potent activity in vivo. Proc Natl Acad Sci USA. 2021. 10.1073/pnas.2108281118. 34531324 10.1073/pnas.2108281118 PMC8463862

[60] Eckhardt J, Xing Z, Subramanian V, Vaidya A, Cutler S. Robotic imaging and machine learning analysis of seed germination: dissecting the Influence of ABA and DOG1 on germination uniformity plant biology. BioRxiv. 2024. 10.1101/2024.05.10.593629v1.full.pdf.38076945

[61] Mega R, Abe F, Kim J-S, Tsuboi Y, Tanaka K, Kobayashi H, et al. Tuning water-use efficiency and drought tolerance in wheat using abscisic acid receptors. Nat Plants. 2019;5:153–9. 30737511 10.1038/s41477-019-0361-8

